# Unmet need in severe, uncontrolled asthma: can anti-TSLP therapy with tezepelumab provide a valuable new treatment option?

**DOI:** 10.1186/s12931-020-01505-x

**Published:** 2020-10-15

**Authors:** Andrew Menzies-Gow, Michael E. Wechsler, Chris E. Brightling

**Affiliations:** 1 Royal Brompton Hospital, Sydney Street, London, SW3 6NP UK; 2 National Jewish Health, Denver, CO USA; 3 University of Leicester, Leicester, UK

**Keywords:** Asthma, Biologics, Burden of illness, Phenotype, Tezepelumab, Thymic stromal lymphopoietin

## Abstract

Despite treatment with standard-of-care medications, including currently available biologic therapies, many patients with severe asthma have uncontrolled disease, which is associated with a high risk of hospitalization and high healthcare costs. Biologic therapies approved for severe asthma have indications limited to patients with either eosinophilic or allergic phenotypes; there are currently no approved biologics for patients with eosinophil-low asthma. Furthermore, existing biologic treatments decrease exacerbation rates by approximately 50% only, which may be because they target individual, downstream elements of the asthma inflammatory response, leaving other components untreated. Targeting an upstream mediator of the inflammatory response may have a broader effect on airway inflammation and provide more effective asthma control. One such potential target is thymic stromal lymphopoietin (TSLP), an epithelial-derived cytokine released in response to multiple triggers associated with asthma exacerbations, such as viruses, allergens, pollutants and other airborne irritants. Mechanistic studies indicate that TSLP drives eosinophilic (including allergic) inflammation, neutrophilic inflammation and structural changes to the airway in asthma through actions on a wide variety of adaptive and innate immune cells and structural cells. Tezepelumab is a first-in-class human monoclonal antibody that blocks the activity of TSLP. In the phase 2b PATHWAY study (NCT02054130), tezepelumab reduced asthma exacerbations by up to 71% compared with placebo in patients with severe, uncontrolled asthma across the spectrum of inflammatory phenotypes, and improved lung function and asthma control. Phase 3 trials of tezepelumab are underway. NAVIGATOR (NCT03347279), a pivotal exacerbation study, aims to assess the potential efficacy of tezepelumab further in patients with a broad range of severe asthma phenotypes, including those with low blood eosinophil counts. SOURCE (NCT03406078) aims to evaluate the oral corticosteroid-sparing potential of tezepelumab. DESTINATION (NCT03706079) is a long-term extension study. In addition, an ongoing phase 2 bronchoscopy study, CASCADE (NCT03688074), aims to evaluate the effect of tezepelumab on airway inflammation and airway remodelling in patients across the spectrum of type 2 airway inflammation. Here, we summarize the unmet therapeutic need in severe asthma and the current treatment landscape, discuss the rationale for targeting TSLP in severe asthma therapy and describe the current development status of tezepelumab.

## Background

Asthma is a highly prevalent inflammatory disease of the airways, with current estimates putting the number of individuals with the condition at 339 million worldwide [1]. While the majority of these individuals have mild disease, approximately 5–10% have severe asthma [2, 3]. Many patients with severe asthma have disease that remains uncontrolled despite standard-of-care therapy [2, 4], which severely impacts their health-related quality of life (HRQoL) owing to persistent symptoms and frequent and life-threatening exacerbations [5, 6]. Patients with severe asthma have a high rate of healthcare resource use, accounting for twice as many asthma-related hospitalizations as those with non-severe disease [7], and incur approximately two-threefold higher asthma-related healthcare and medication costs [7, 8]. Comorbidities of severe asthma are common and include chronic rhinosinusitis, nasal polyposis, allergic rhinitis and gastroesophageal reflux disease [9]. Furthermore, 20–60% of patients with severe or uncontrolled asthma may receive long-term oral corticosteroid (OCS) therapy [10], which is associated with a range of side effects including infections and cardiovascular, metabolic, psychiatric, ocular, gastrointestinal and bone-related complications [11].

The inflammation associated with asthma is heterogeneous, with considerable variation in the profiles of upregulated immune cells and biomarkers. From these profiles, several inflammatory phenotypes of asthma can be designated. The most common phenotypes involve eosinophilic (including allergic) type 2 (T2) inflammation. Eosinophilic phenotypes are characterized by elevated blood eosinophil counts, typically defined as at least 150 or 300 cells/µL, and/or elevated sputum eosinophil levels of at least 2–3% [12]. In the case of allergic asthma, patients also exhibit a serum immunoglobulin (Ig) E level of at least 30 IU/mL, in addition to sensitivity to a perennial aeroallergen and symptoms that are allergy-driven [13]. Fractional exhaled nitric oxide (FeNO) levels may also be elevated in patients with eosinophilic or allergic phenotypes, although there is not yet a consensus on the threshold that constitutes elevated FeNO. Other phenotypes are characterized by predominantly neutrophilic inflammation with normal eosinophil counts, elevated counts of eosinophils and neutrophils (mixed granulocytic inflammation), or by normal or low counts of neutrophils and eosinophils (paucigranulocytic inflammation) [12, 14, 15]. In relation to these phenotypes, it should be noted that corticosteroid therapy reduces eosinophil counts and may also increase neutrophil counts [16]; thus a patient’s apparent phenotype may be a product of treatment choices rather than a true reflection of the underlying disease mechanisms. In addition to the heterogeneity of immune cells and biomarkers, there is variability among patients in other pathophysiological features of asthma, such as airway hyperresponsiveness and remodelling, which may be related to or separate from inflammatory events in the airways [17].

### Current treatment options for severe asthma

The heterogeneity of severe asthma makes management of the disease highly challenging, and intensive treatment regimens involving multiple medications are required. Global Initiative for Asthma (GINA) guidelines (treatment steps 4 and 5) recommend a combination medium-to-high-dose inhaled corticosteroid (ICS) and long-acting β_2_ agonist (LABA) as maintenance therapy to prevent exacerbations and to control symptoms in these patients, with additional relief from a short-acting β_2_ agonist as needed (or an ICS/LABA combination as both maintenance and reliever therapy, in the case of formoterol-containing combinations) [18]. Other controller medications recommended by GINA and European Respiratory Society/American Thoracic Society guidelines for use as potential add-on therapies are inhaled tiotropium (a long-acting muscarinic antagonist; at GINA steps 4 or 5), leukotriene receptor antagonists (at step 4) and macrolides (at step 5) [18, 19]. However, many patients have an insufficient response to these treatments and so are prescribed frequent bursts of OCS [10], although the side effect profile of OCS limits the dose and makes chronic administration non-ideal. Furthermore, some patients have steroid-refractory disease. At GINA step 5, it is recommended that patients are assessed for eligibility for biologic therapies, which are indicated as additional controllers for specific phenotypes of severe asthma [18, 19]. Eligibility for these therapies is partly determined by meeting specific inflammatory biomarker thresholds (and, by extension, phenotypes) for which efficacy of the particular biologic treatment was demonstrated in clinical trials.

Currently available biologics for severe asthma comprise anti-IgE (omalizumab), anti-IL-5 (mepolizumab, reslizumab), anti-IL-5 receptor α (benralizumab) and anti-IL-4 receptor α (dupilumab, which blocks the IL-4 and IL-13 pathways) monoclonal antibodies. These therapies are generally indicated for patients with eosinophilic or allergic asthma phenotypes; to date, there are no approved biologic treatments for patients with confirmed eosinophil-low asthma (in the absence of eosinophil-lowering systemic corticosteroid therapy). In the pivotal studies of the currently approved biologics, exacerbation rates were reduced by 48–59% with the most efficacious dose regimen versus placebo [20–24]. Improvements in lung function and symptom scores were inconsistent, with only some individuals experiencing clinically significant improvements [25–28]. A possible explanation for this lack of complete efficacy may be that these biologics target individual, downstream elements of the asthma inflammatory response, leaving other components untreated. Targeting an upstream initiator and mediator of the inflammatory response may, therefore, have a broader effect on airway inflammation and provide more effective asthma control, including in patients with eosinophil-low phenotypes. One such potential target is thymic stromal lymphopoietin (TSLP).

### Thymic stromal lymphopoietin

TSLP is a member of the class of cytokines known as the alarmins, which also includes IL-25 and IL-33. It is primarily expressed by the airway epithelium and released in response to environmental insults such as allergens, viruses, bacteria, pollutants and physical injury, instigating a range of downstream inflammatory processes [29, 30]. There is strong evidence that TSLP dysregulation plays an important role in the pathophysiology of asthma.

TSLP expression is increased in the airways of patients with asthma compared with healthy individuals [31–34], correlating with disease severity and impairment of lung function [31, 33, 35, 36]. Furthermore, genome-wide association studies have identified associations between asthma risk and single-nucleotide polymorphisms in the *TSLP* gene [37–40].

Following its release from the epithelium, TSLP drives allergic and non-allergic eosinophilic inflammation, leading to various features of asthma pathophysiology [41]. TSLP activates dendritic cells in response to allergen exposure, inducing differentiation of naïve T cells to Th2 cells, which produce IL-4, IL-5 and IL-13. This leads to IgE switching in B cells, degranulation of mast cells, airway eosinophilia, mucus hypersecretion from goblet cells and smooth muscle contraction resulting in airway hyperresponsiveness [42, 43]. Following exposure to viruses, bacteria, pollutants and other insults, TSLP (as well as IL-33 and IL-25) activates group 2 innate lymphoid cells (ILC2s), which produce IL-5 and IL-13 [44]. In addition to its T2-related effects, there is growing evidence that TSLP plays a role in non-T2 processes involving both immune and structural cells. TSLP is thought to play a role in neutrophilic airway inflammation by activating dendritic cells to induce polarization of naïve T cells towards a Th17 phenotype, which subsequently release IL-17 [45, 46]. TSLP also promotes airway remodelling by stimulating airway smooth muscle cell migration [47], facilitating cross-talk between mast cells and airway smooth muscle cells [48] and stimulating fibroblast cells to produce collagen [49, 50]. The myriad effects of TSLP in asthma therefore make it an attractive therapeutic target.

### Tezepelumab

**Fig. 1:**
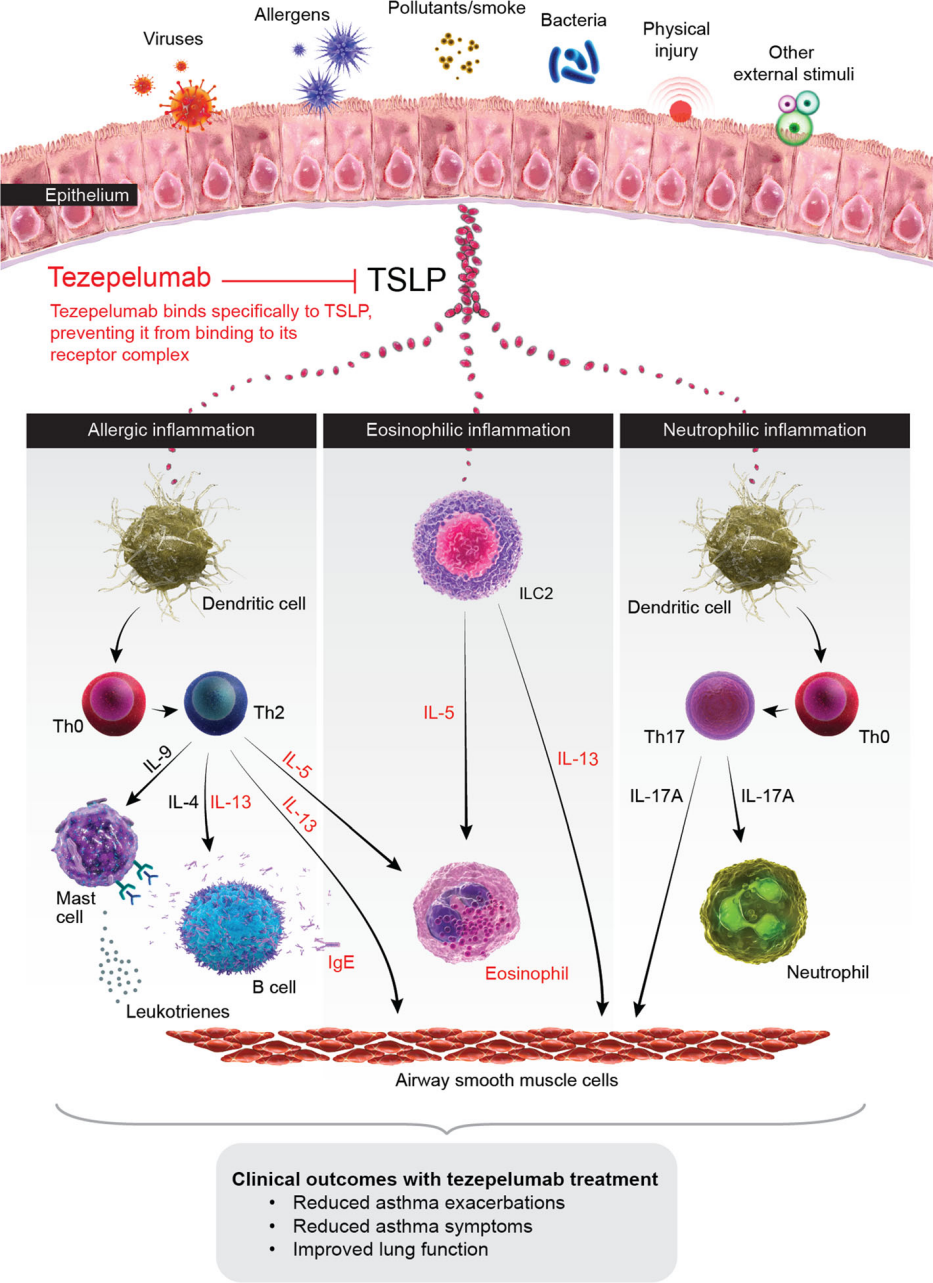
Mechanism of action by which tezepelumab improves clinical outcomes in patients with severe asthma. TSLP is released from the airway epithelium in response to insults such as viruses, allergens and pollutants, triggering an inflammatory cascade. Overexpression of TSLP can result in pathologic inflammation that can lead to asthma exacerbations, symptoms, and physiological effects such as bronchoconstriction and airway hyperresponsiveness and remodelling. Tezepelumab specifically blocks TSLP from binding to its heterodimeric receptor, thereby inhibiting the production of various inflammatory cytokines and cell types. Treatment with tezepelumab has thus far been shown to reduce eosinophils, IgE, IL-5, IL-13 and FeNO. *FeNO*, fractional exhaled nitric oxide; *IgE*, immunoglobulin E; *IL*, interleukin; *ILC2*, type 2 innate lymphoid cell; *Th*, T-helper; *TSLP*, thymic stromal lymphopoietin

Tezepelumab is a human monoclonal antibody (IgG2λ) that selectively blocks TSLP from interacting with its heterodimeric receptor (Fig. 1) [51–53]. In a phase 1b, proof-of-concept study in patients with mild allergic asthma (ClinicalTrials.gov identifier NCT01405963), tezepelumab attenuated asthmatic responses to allergen challenge and reduced biomarkers of inflammation compared with placebo [51]. In the tezepelumab group, blood eosinophil counts began to decline at 2 weeks post-dosing and reached normal levels by 4 weeks, while sputum eosinophils reached normal levels by 6 weeks (the first time point measured). FeNO levels improved significantly 1 week after the first dose. The phase 2b PATHWAY study, a multicentre, randomized, double-blind, placebo-controlled trial (NCT02054130) evaluated the efficacy and safety of tezepelumab as an add-on therapy for patients with severe, uncontrolled asthma, who were receiving medium-to-high-dose ICS and a LABA with or without OCS and additional asthma controllers [52]. Tezepelumab treatment was associated with significant reductions in annualized exacerbation rates of up to 71% versus placebo. Subgroup analyses showed that these reductions were significant irrespective of patient phenotype, as measured by baseline blood eosinophil counts (< 150 cells/µL, ≥ 150 cells/µL, < 300 cells/µL or ≥ 300 cells/µL), as well as by FeNO and IgE levels, suggesting that tezepelumab provided similar efficacy in patients across the spectrum of inflammatory phenotypes [54]. Compared with placebo, tezepelumab also reduced asthma-exacerbation-related hospitalizations and improved lung function, asthma control and patient HRQoL [52]. Further analyses of the PATHWAY data showed that tezepelumab reduced blood eosinophil counts, FeNO levels, serum IgE and cytokines including IL-5 and IL-13 [52, 55]. Of the currently approved biologics, only anti-IgE has been shown to reduce FeNO levels, serum free IgE and blood or sputum eosinophil counts, although not to the same extent as anti-TSLP therapy [56]. The safety profile of tezepelumab in PATHWAY was acceptable, with a similar overall frequency of adverse events (including serious adverse events) in patients receiving tezepelumab and those receiving placebo; the most common adverse events were consistent with those expected in a patient population with severe asthma [52]. A low incidence of anti-drug antibodies was observed in patients treated with tezepelumab, without any apparent clinical impact. The observed efficacy of tezepelumab in a broad range of patients with severe asthma in PATHWAY led to it being designated a ‘breakthrough therapy’ by the US Food and Drug Administration in 2019 for patients with severe asthma without an eosinophilic phenotype, who are receiving ICS/LABA and additional asthma controllers with or without OCS [57].

Further trials of tezepelumab in patients with asthma are underway (Table 1). NAVIGATOR (NCT03347279) [58] is a pivotal phase 3 study assessing the potential efficacy of tezepelumab in adults and adolescents with severe, uncontrolled asthma spanning a broad range of phenotypes, aiming to build on the findings of PATHWAY. The recruited participants comprise roughly equal proportions of patients with baseline blood eosinophil counts of less than 300 cells/µL and at least 300 cells/µL; approximately 25% of patients have an eosinophil count of less than 150 cells/µL or of greater than 450 cells/µL. The primary efficacy endpoint is the annualized asthma exacerbation rate during the 52-week treatment period in the overall patient population, and this will also be assessed separately in patients with baseline blood eosinophil counts below 300 cells/µL. Key secondary endpoints of NAVIGATOR assess lung function, asthma control and HRQoL. SOURCE (NCT03406078) [59] is a phase 3 study that aims to evaluate the OCS-sparing potential of tezepelumab in adults with OCS-dependent asthma. Following OCS dose optimization, the 48-week treatment period comprises: a 4-week induction phase during which tezepelumab is introduced; a 36-week OCS reduction phase during which the OCS dose is tapered (dependent on the patient continuing to meet asthma control criteria); and an 8-week maintenance phase in which patients continue on their final OCS dose. The primary endpoint is the percentage reduction from baseline to week 48 in the prescribed daily OCS maintenance dose while not losing asthma control. DESTINATION (NCT03706079) [60] is a long-term extension of NAVIGATOR and SOURCE that addresses the safety and tolerability of tezepelumab for up to 2 years’ treatment. In addition to these phase 3 studies, the phase 2 mechanistic study CASCADE (NCT03688074) [61] aims to evaluate the effect of tezepelumab on airway inflammation and airway remodelling in patients spanning the spectrum of T2 airway inflammation, as determined by epithelial molecular phenotyping. The primary endpoint is the change from baseline to week 28 in airway submucosal inflammatory cells (eosinophils, neutrophils, T cells and mast cells) from bronchoscopic biopsies. Secondary endpoints include assessments of reticular basement membrane thickening and airway epithelial integrity.

**Table 1:** Ongoing studies of tezepelumab in patients with asthma and healthy volunteers

ClinicalTrials.gov identifier	Estimated start and completion dates	Patient population	Phase	Primary outcome
NCT02698501 (UPSTREAM)	2016–2019	40 adults with asthma requiring ICS (± LABA)	2	Mannitol PD_15_
NCT03989544 (PATH-BRIDGE)	2019–2019	315 healthy adults	1	Pharmacokinetics of SC administration via accessorized pre-filled syringe or autoinjector compared with vial and syringe
NCT03968978 (PATH-HOME)	2019–2020	216 adults and adolescents with severe asthma	3	Successful SC administration via accessorized pre-filled syringe or autoinjector at home versus in the clinic
NCT03347279 (NAVIGATOR)	2019–2020	1038 adults and adolescents with severe, uncontrolled asthma, taking medium- to high-dose ICS and at least one additional asthma controller with or without OCS	3	Annualized asthma exacerbation rate
NCT03406078 (SOURCE)	2018–2020	150 adults with oral corticosteroid-dependent asthma (Americas, Europe)	3	Reduction in daily OCS dose
NCT03688074 (CASCADE)	2018–2020	116 adults with inadequately controlled moderate-to-severe asthma, taking ICS and at least one additional asthma controller	2	Number of airway submucosal inflammatory cells/mm^2^ of bronchoscopic biopsies
NCT03706079 (DESTINATION)	2019–2022	966 adults and adolescents with severe, uncontrolled asthma	3	Exposure-adjusted incidence of adverse events and serious adverse events
NCT04048343 (NOZOMI)	2019–2021	66 Japanese adults and adolescents with inadequately controlled severe asthma	3	Rate of adverse events
NCT03927157 (DIRECTION)	2019–2023	396 Chinese adults with severe, uncontrolled asthma taking medium- to high-dose ICS and at least one additional asthma controller with or without OCS	3	Annualized asthma exacerbation rate

*ICS*  Inhaled corticosteroids*, LABA* Long-acting β_2_ agonist*, OCS* Oral corticosteroids*, PD*_*15*_ Provoking dose to induce a 15% fall in forced expiratory volume in 1 s*, SC*  Subcutaneous

## Conclusions

The disease burden of patients with severe asthma is considerable and has yet to be fully addressed with available treatment options, including biologic therapies. In particular, there are currently no approved therapies for patients with eosinophil-low asthma. Blocking the activity of TSLP has the potential to enact broader effects on airway inflammation than are achieved with current biologic therapies owing to its position at the top of the inflammatory cascade, from which it mediates a wide range of downstream processes that drive eosinophilic and neutrophilic inflammation, as well as structural changes to the airway. Clinical trials of TSLP blockade with tezepelumab completed to date have yielded promising results in patients with a variety of asthma phenotypes, who experienced significant reductions in exacerbation rates and improvements in lung function, symptom control and HRQoL. Studies in progress will provide further evidence of whether tezepelumab can be an effective treatment for a broad population of patients with severe asthma.

## Data Availability

Not applicable.

## Abbreviations

FeNO: Fractional exhaled nitric oxide
GINA: Global Initiative for Asthma
HRQoL: Health-related quality of life
ICS: Inhaled corticosteroids
Ig: Immunoglobulin
IL: Interleukin
ILC2: Group 2 innate lymphoid cell
LABA: Long-acting β_2_ agonist
OCS: Oral corticosteroids
SABA: Short-acting β_2_ agonist
T2: Type 2
TSLP: Thymic stromal lymphopoietin

## References

[1] Global Asthma Network. Global Asthma Report 2018. Available from: https://www.globalasthmareport.org/. Accessed 12 March 2020.

[2] Chung KF, Wenzel SE, Brozek JL, Bush A, Castro M, Sterk PJ, et al. International ERS/ATS guidelines on definition, evaluation and treatment of severe asthma. Eur Respir J. 2014;43(2):343–73. 10.1183/09031936.00202013 24337046

[3] Backman H, Jansson SA, Stridsman C, Eriksson B, Hedman L, Eklund BM, et al. Severe asthma - A population study perspective. Clin Exp Allergy. 2019;49(6):819–28. 10.1111/cea.13378 30817038

[4] Chen S, Golam S, Myers J, Bly C, Smolen H, Xu X. Systematic literature review of the clinical, humanistic, and economic burden associated with asthma uncontrolled by GINA Steps 4 or 5 treatment. Curr Med Res Opin. 2018;34(12):2075–88. 10.1080/03007995.2018.1505352 30047292

[5] Chen H, Gould MK, Blanc PD, Miller DP, Kamath TV, Lee JH, et al. Asthma control, severity, and quality of life: quantifying the effect of uncontrolled disease. J Allergy Clin Immunol. 2007;120(2):396–402. 10.1016/j.jaci.2007.04.040 17561244

[6] McDonald VM, Hiles SA, Jones KA, Clark VL, Yorke J. Health-related quality of life burden in severe asthma. Med J Aust. 2018;209(S2):S28–33. 10.5694/mja18.00207 30453870

[7] Chastek B, Korrer S, Nagar SP, Albers F, Yancey S, Ortega H, et al. Economic burden of illness among patients with severe asthma in a managed care setting. J Manag Care Spec Pharm. 2016;22(7):848–61. 10.18553/jmcp.2016.22.7.848 PMC10397901 27348285

[8] Zeiger RS, Schatz M, Dalal AA, Qian L, Chen W, Ngor EW, et al. Utilization and costs of severe uncontrolled asthma in a managed-care setting. J Allergy Clin Immunol Pract. 2016;4(1):120–9. 10.1016/j.jaip.2015.08.003 26439182

[9] Porsbjerg C, Menzies-Gow A. Co-morbidities in severe asthma: clinical impact and management. Respirology. 2017;22(4):651–61. 10.1111/resp.13026 28328160

[10] Bleecker ER, Menzies-Gow AN, Price DB, Bourdin A, Sweet S, Martin AL, et al. Systematic literature review of systemic corticosteroid use for asthma management. Am J Respir Crit Care Med. 2020;201(3):276–93. 10.1164/rccm.201904-0903SO PMC6999108 31525297

[11] Lefebvre P, Duh MS, Lafeuille MH, Gozalo L, Desai U, Robitaille MN, et al. Acute and chronic systemic corticosteroid-related complications in patients with severe asthma. J Allergy Clin Immunol. 2015;136(6):1488–95. 10.1016/j.jaci.2015.07.046 26414880

[12] Carr TF, Zeki AA, Kraft M. Eosinophilic and noneosinophilic asthma. Am J Respir Crit Care Med. 2018;197(1):22–37. 10.1164/rccm.201611-2232PP PMC5765385 28910134

[13] Carr TF, Kraft M. Use of biomarkers to identify phenotypes and endotypes of severe asthma. Ann Allergy Asthma Immunol. 2018;121(4):414–20. 10.1016/j.anai.2018.07.029 30059792

[14] Moore WC, Hastie AT, Li X, Li H, Busse WW, Jarjour NN, et al. Sputum neutrophil counts are associated with more severe asthma phenotypes using cluster analysis. J Allergy Clin Immunol. 2014;133(6):1557–63. 10.1016/j.jaci.2013.10.011 PMC4040309 24332216

[15] Tliba O, Panettieri RA (2019). Paucigranulocytic asthma: uncoupling of airway obstruction from inflammation. J Allergy Clin Immunol.

[16] Cowan DC, Cowan JO, Palmay R, Williamson A, Taylor DR (2010). Effects of steroid therapy on inflammatory cell subtypes in asthma. Thorax.

[17] Saglani S, Lloyd CM. Novel concepts in airway inflammation and remodelling in asthma. Eur Respir J. 2015;46(6):1796–804. 10.1183/13993003.01196-2014 26541520

[18] Global Initiative for Asthma. Global strategy for asthma management and prevention. Updated 2019. Available from https://ginasthma.org/gina-reports/. Accessed 27 Jan 2020.

[19] Holguin F, Cardet JC, Chung KF, Diver S, Ferreira DS, Fitzpatrick A, et al. Management of severe asthma: a European Respiratory Society/American Thoracic Society guideline. Eur Respir J. 2020;55(1):1900588. 10.1183/13993003.00588-2019 31558662

[20] Busse W, Corren J, Lanier BQ, McAlary M, Fowler-Taylor A, Cioppa GD, et al. Omalizumab, anti-IgE recombinant humanized monoclonal antibody, for the treatment of severe allergic asthma. J Allergy Clin Immunol. 2001;108(2):184–90. 10.1067/mai.2001.117880 11496232

[21] Pavord ID, Korn S, Howarth P, Bleecker ER, Buhl R, Keene ON, et al. Mepolizumab for severe eosinophilic asthma (DREAM): a multicentre, double-blind, placebo-controlled trial. Lancet. 2012;380(9842):651–9. 10.1016/S0140-6736(12)60988-X 22901886

[22] Castro M, Zangrilli J, Wechsler ME, Bateman ED, Brusselle GG, Bardin P (2015). Reslizumab for inadequately controlled asthma with elevated blood eosinophil counts: results from two multicentre, parallel, double-blind, randomised, placebo-controlled, phase 3 trials. Lancet Respir Med.

[23] Bleecker ER, FitzGerald JM, Chanez P, Papi A, Weinstein SF, Barker P, et al. Efficacy and safety of benralizumab for patients with severe asthma uncontrolled with high-dosage inhaled corticosteroids and long-acting beta2-agonists (SIROCCO): a randomised, multicentre, placebo-controlled phase 3 trial. Lancet. 2016;388(10056):2115–27. 10.1016/S0140-6736(16)31324-1 27609408

[24] Castro M, Corren J, Pavord ID, Maspero J, Wenzel S, Rabe KF, et al. Dupilumab efficacy and safety in moderate-to-severe uncontrolled asthma. N Engl J Med. 2018;378(26):2486–96. 10.1056/NEJMoa1804092 29782217

[25] Normansell R, Walker S, Milan SJ, Walters EH, Nair P. Omalizumab for asthma in adults and children. Cochrane Database Syst Rev. 2014(1):CD003559. 10.1002/14651858.CD003559.pub4 PMC10981784 24414989

[26] Farne HA, Wilson A, Powell C, Bax L, Milan SJ. Anti-IL5 therapies for asthma. Cochrane Database Syst Rev. 2017;9:CD010834. 10.1002/14651858.CD010834.pub3 PMC6483800 28933516

[27] Zayed Y, Kheiri B, Banifadel M, Hicks M, Aburahma A, Hamid K, et al. Dupilumab safety and efficacy in uncontrolled asthma: a systematic review and meta-analysis of randomized clinical trials. J Asthma. 2019;56(10):1110–19. 10.1080/02770903.2018.1520865 30273510

[28] Xiong XF, Zhu M, Wu HX, Fan LL, Cheng DY. Efficacy and safety of dupilumab for the treatment of uncontrolled asthma: a meta-analysis of randomized clinical trials. Respir Res. 2019;20(1):108. 10.1186/s12931-019-1065-3 PMC6544936 31151443

[29] Mitchell PD, O’Byrne PM. Epithelial-derived cytokines in asthma. Chest. 2017;151(6):1338–44. 10.1016/j.chest.2016.10.042 27818325

[30] Corren J, Ziegler SF (2019). TSLP: from allergy to cancer. Nat Immunol.

[31] Ying S, O’Connor B, Ratoff J, Meng Q, Mallett K, Cousins D, et al. Thymic stromal lymphopoietin expression is increased in asthmatic airways and correlates with expression of Th2-attracting chemokines and disease severity. J Immunol. 2005;174(12):8183–90. 10.4049/jimmunol.174.12.8183 15944327

[32] Ying S, O’Connor B, Ratoff J, Meng Q, Fang C, Cousins D, et al. Expression and cellular provenance of thymic stromal lymphopoietin and chemokines in patients with severe asthma and chronic obstructive pulmonary disease. J Immunol. 2008;181(4):2790–8. 10.4049/jimmunol.181.4.2790 18684970

[33] Shikotra A, Choy DF, Ohri CM, Doran E, Butler C, Hargadon B, et al. Increased expression of immunoreactive thymic stromal lymphopoietin in patients with severe asthma. J Allergy Clin Immunol. 2012;129(1):104–11. 10.1016/j.jaci.2011.08.031 21975173

[34] Bleck B, Kazeros A, Bakal K, Garcia-Medina L, Adams A, Liu M (2015). Coexpression of type 2 immune targets in sputum-derived epithelial and dendritic cells from asthmatic subjects. J Allergy Clin Immunol.

[35] Bjerregaard A, Laing IA, Poulsen N, Backer V, Sverrild A, Fally M (2017). Characteristics associated with clinical severity and inflammatory phenotype of naturally occurring virus-induced exacerbations of asthma in adults. Respir Med.

[36] Li Y, Wang W, Lv Z, Li Y, Chen Y, Huang K (2018). Elevated expression of IL-33 and TSLP in the airways of human asthmatics in vivo: A potential biomarker of severe refractory disease. J Immunol.

[37] He JQ, Hallstrand TS, Knight D, Chan-Yeung M, Sandford A, Tripp B (2009). A thymic stromal lymphopoietin gene variant is associated with asthma and airway hyperresponsiveness. J Allergy Clin Immunol.

[38] Moffatt MF, Gut IG, Demenais F, Strachan DP, Bouzigon E, Heath S (2010). A large-scale, consortium-based genomewide association study of asthma. N Engl J Med.

[39] Torgerson DG, Ampleford EJ, Chiu GY, Gauderman WJ, Gignoux CR, Graves PE (2011). Meta-analysis of genome-wide association studies of asthma in ethnically diverse North American populations. Nat Genet.

[40] Moorehead A, Hanna R, Heroux D, Neighbour H, Sandford A, Gauvreau GM (2020). A thymic stromal lymphopoietin polymorphism may provide protection from asthma by altering gene expression. Clin Exp Allergy.

[41] Gauvreau GM, Sehmi R, Ambrose CS, Griffiths JM. Thymic stromal lymphopoietin: its role and potential as a therapeutic target in asthma. Expert Opin Ther Targets. 2020:1–16. https://pubmed.ncbi.nlm.nih.gov/32567399/. 10.1080/14728222.2020.1783242 32567399

[42] Watson B, Gauvreau GM. Thymic stromal lymphopoietin: a central regulator of allergic asthma. Expert Opin Ther Targets. 2014;18(7):771–85. 10.1517/14728222.2014.915314 24930783

[43] Zhang Y, Zhou B. Functions of thymic stromal lymphopoietin in immunity and disease. Immunol Res. 2012;52(3):211–23. 10.1007/s12026-012-8264-z PMC3350568 22274860

[44] Klose CS, Artis D. Innate lymphoid cells as regulators of immunity, inflammation and tissue homeostasis. Nat Immunol. 2016;17(7):765–74. 10.1038/ni.3489 27328006

[45] Tanaka J, Watanabe N, Kido M, Saga K, Akamatsu T, Nishio A (2009). Human TSLP and TLR3 ligands promote differentiation of Th17 cells with a central memory phenotype under Th2-polarizing conditions. Clin Exp Allergy.

[46] Gao H, Ying S, Dai Y. Pathological roles of neutrophil-mediated inflammation in asthma and its potential for therapy as a target. J Immunol Res. 2017;2017:3743048. 10.1155/2017/3743048 PMC5735647 29359169

[47] Redhu NS, Shan L, Movassagh H, Gounni AS. Thymic stromal lymphopoietin induces migration in human airway smooth muscle cells. Sci Rep. 2013;3:2301. 10.1038/srep02301 PMC3725475 23892442

[48] Kaur D, Doe C, Woodman L, Heidi Wan WY, Sutcliffe A, Hollins F, et al. Mast cell-airway smooth muscle crosstalk: the role of thymic stromal lymphopoietin. Chest. 2012;142(1):76–85. 10.1378/chest.11-1782 PMC3418864 22052771

[49] Cao L, Liu F, Liu Y, Liu T, Wu J, Zhao J, et al. TSLP promotes asthmatic airway remodeling via p38-STAT3 signaling pathway in human lung fibroblast. Exp Lung Res. 2018;44(6):288–301. 10.1080/01902148.2018.1536175 30428724

[50] Wu J, Liu F, Zhao J, Wei Y, Lv J, Dong F, et al. Thymic stromal lymphopoietin promotes asthmatic airway remodelling in human lung fibroblast cells through STAT3 signalling pathway. Cell Biochem Funct. 2013;31(6):496–503. 10.1002/cbf.2926 23192865

[51] Gauvreau GM, O’Byrne PM, Boulet LP, Wang Y, Cockcroft D, Bigler J, et al. Effects of an anti-TSLP antibody on allergen-induced asthmatic responses. N Engl J Med. 2014;370(22):2102–10. 10.1056/NEJMoa1402895 24846652

[52] Corren J, Parnes JR, Wang L, Mo M, Roseti SL, Griffiths JM, et al. Tezepelumab in adults with uncontrolled asthma. N Engl J Med. 2017;377(10):936–46. 10.1056/NEJMoa1704064 28877011

[53] Sakamoto K, Matsuki S, Irie S, Uchida N, Hayashi N, Horiuchi M, et al. A phase 1, randomized, placebo-controlled study to evaluate the safety, tolerability, pharmacokinetics, and immunogenicity of subcutaneous tezepelumab in healthy Japanese men. Clin Pharmacol Drug Dev. 2020. 10.1002/cpdd.775. 10.1002/cpdd.775 PMC7586988 31960624

[54] Corren J, Garcia Gil E, Parnes J, Pham T, Griffiths J. Tezepelumab treatment effect on annualized rate of exacerbations by baseline biomarkers in uncontrolled severe asthma patients: phase 2b PATHWAY study. Am J Respir Crit Care Med. 2019;199:A2621.

[55] Pham T-H, Ren P, Parnes JR, Griffiths JM. Tezepelumab reduces multiple key inflammatory biomarkers in patients with severe, uncontrolled asthma in the phase 2b PATHWAY study. Am J Respir Crit Care Med. 2019;199:A2677.

[56] Upham JW, Jurak LM. How do biologicals and other novel therapies effect clinically used biomarkers in severe asthma? Clin Exp Allergy. 2020;50(9):994–1006. 10.1111/cea.13694 32569412

[57] AstraZeneca. Tezepelumab granted Breakthrough Therapy Designation by US FDA. Available from: https://www.astrazeneca.com/media-centre/press-releases/2018/tezepelumab-granted-breakthrough-therapy-designation-by-us-fda-07092018.html. Accessed 5 Mar 2020.

[58] ClinicalTrials.gov. Study to evaluate tezepelumab in adults and adolescents with severe uncontrolled asthma (NAVIGATOR). Available from: https://clinicaltrials.gov/ct2/show/NCT03347279. Accessed 5 Mar 2020.

[59] ClinicalTrials.gov. Study to evaluate the efficacy and safety of tezepelumab in reducing oral corticosteroid use in adults with oral corticosteroid dependent asthma (SOURCE). Available from: https://clinicaltrials.gov/ct2/show/NCT03406078. Accessed 5 Mar 2020. 10.1186/s12931-020-01503-z PMC7550846 33050928

[60] ClinicalTrials.gov. Extension study to evaluate the safety and tolerability of tezepelumab in adults and adolescents with severe, uncontrolled asthma (DESTINATION). Available from: https://clinicaltrials.gov/ct2/show/NCT03706079. Accessed 5 Mar 2020.

[61] ClinicalTrials.gov. Study to evaluate tezepelumab on airway inflammation in adults with uncontrolled asthma (CASCADE). Available from: https://clinicaltrials.gov/ct2/show/NCT03688074. Accessed 5 Mar 2020.

